# Chrysin-based supramolecular cyclodextrin-calixarene drug delivery system: a novel approach for attenuating cardiac fibrosis in chronic diabetes

**DOI:** 10.3389/fphar.2023.1332212

**Published:** 2023-12-18

**Authors:** Maria Consiglia Trotta, Hildegard Herman, Alina Ciceu, Bianca Mladin, Marcel Rosu, Caterina Claudia Lepre, Marina Russo, Ildikó Bácskay, Ferenc Fenyvesi, Raffaele Marfella, Anca Hermenean, Cornel Balta, Michele D’Amico

**Affiliations:** ^1^ Department of Experimental Medicine, University of Campania “Luigi Vanvitelli”, Naples, Italy; ^2^ “Aurel Ardelean” Institute of Life Sciences, Vasile Goldis Western University of Arad, Arad, Romania; ^3^ PhD Course in Translational Medicine, University of Campania “Luigi Vanvitelli”, Naples, Italy; ^4^ PhD Course in National Interest in Public Administration and Innovation for Disability and Social Inclusion, Department of Mental, Physical Health and Preventive Medicine, University of Campania “Luigi Vanvitelli”, Naples, Italy; ^5^ School of Pharmacology and Clinical Toxicology, University of Campania “Luigi Vanvitelli”, Naples, Italy; ^6^ Department of Molecular and Nanopharmaceutics, Faculty of Pharmacy, University of Debrecen, Debrecen, Hungary; ^7^ Institute of Healthcare Industry, University of Debrecen, Debrecen, Hungary; ^8^ Department of Advanced Medical and Surgical Sciences, University of Campania “Luigi Vanvitelli”, Naples, Italy; ^9^ Department of Histology, Faculty of Medicine, Vasile Goldis Western University of Arad, Arad, Romania

**Keywords:** chrysin, cyclodextrin, calixarene, drug delivery system, cardiac fibrosis, chronic diabetes

## Abstract

**Introduction:** Cardiac fibrosis is strongly induced by diabetic conditions. Both chrysin (CHR) and calixarene OTX008, a specific inhibitor of galectin 1 (Gal-1), seem able to reduce transforming growth factor beta (TGF-β)/SMAD pro-fibrotic pathways, but their use is limited to their low solubility. Therefore, we formulated a dual-action supramolecular system, combining CHR with sulfobutylated β-cyclodextrin (SBECD) and OTX008 (SBECD + OTX + CHR). Here we aimed to test the anti-fibrotic effects of SBECD + OTX + CHR in hyperglycemic H9c2 cardiomyocytes and in a mouse model of chronic diabetes.

**Methods:** H9c2 cardiomyocytes were exposed to normal (NG, 5.5 mM) or high glucose (HG, 33 mM) for 48 h, then treated with SBECD + OTX + CHR (containing OTX008 0.75–1.25–2.5 µM) or the single compounds for 6 days. TGF-β/SMAD pathways, Mitogen-Activated Protein Kinases (MAPKs) and Gal-1 levels were assayed by Enzyme-Linked Immunosorbent Assays (ELISAs) or Real-Time Quantitative Reverse Transcription Polymerase chain reaction (qRT-PCR). Adult CD1 male mice received a single intraperitoneal (i.p.) administration of streptozotocin (STZ) at a dosage of 102 mg/kg body weight. From the second week of diabetes, mice received 2 times/week the following i.p. treatments: OTX (5 mg/kg)-SBECD; OTX (5 mg/kg)-SBECD-CHR, SBECD-CHR, SBECD. After a 22-week period of diabetes, mice were euthanized and cardiac tissue used for tissue staining, ELISA, qRT-PCR aimed to analyse TGF-β/SMAD, extracellular matrix (ECM) components and Gal-1.

**Results:** In H9c2 cells exposed to HG, SBECD + OTX + CHR significantly ameliorated the damaged morphology and reduced TGF-β1, its receptors (TGFβR1 and TGFβR2), SMAD2/4, MAPKs and Gal-1. Accordingly, these markers were reduced also in cardiac tissue from chronic diabetes, in which an amelioration of cardiac remodeling and ECM was evident. In both settings, SBECD + OTX + CHR was the most effective treatment compared to the other ones.

**Conclusion:** The CHR-based supramolecular SBECD-calixarene drug delivery system, by enhancing the solubility and the bioavailability of both CHR and calixarene OTX008, and by combining their effects, showed a strong anti-fibrotic activity in rat cardiomyocytes and in cardiac tissue from mice with chronic diabetes. Also an improved cardiac tissue remodeling was evident. Therefore, new drug delivery system, which could be considered as a novel putative therapeutic strategy for the treatment of diabetes-induced cardiac fibrosis.

## 1 Introduction

Cardiac fibrosis is a prominent outcome in heart-related disorders, exhibited by various cardiac regions (Tian et al., 2017; Ytrehus et al., 2018). The main cells involved in development of cardiac fibrosis are activated myofibroblasts originating from the epicardium (Tao et al., 2013), the endocardium (Wessels et al., 2012), and the cardiac neural crest (Ali et al., 2014a). Other cells, like macrophages and endothelial cells, also participate in cardiac fibrogenesis via unique molecular pathways (Jiang et al., 2021).

The activation of cardiac myofibroblasts leads to an increased deposition the extracellular matrix (ECM), which amplifies cardiac dysfunction, leads to interstitial fibrosis and can consequently cause heart failure (González et al., 2018; Jiang et al., 2021). However, although cardiac fibrosis is frequently associated with myocardial infarction, it characterizes also idiopathic dilated cardiomyopathy, hypertensive heart disease, and diabetic hypertrophic cardiomyopathy (Jellis et al., 2010; Disertori et al., 2017). Particularly, the first asymptomatic stage of diabetic cardiomyopathy is characterized by myocardial fibrosis, worsened by hyperglycaemia (Jia et al., 2018).

To this regard, Galectin 1 (Gal-1) protein has recently emerged as a promising target for treating diabetes-induced fibrosis. Indeed, it has been found upregulated in kidneys from mice with type 1 and type 2 diabetes (Kuo et al., 2020), contributing to the progression of kidney fibrosis (Liu et al., 2015). Similarly, Gal-1 increase has been shown in cardiac disorders promoted by fibrotic processes, such as heart failure and acute myocardial infarction (Talman and Ruskoaho, 2016; Seropian et al., 2018). Interestingly, the Gal-1 inhibition by OTX008 compound has been effective in counteracting the buildup of Gal-1 under high glucose conditions. Indeed, a previous study reproducing *in vitro* diabetic retinopathy reported a reduction of Transforming Growth Factor beta 1 (TGF-β1) in human retinal pigment epithelial cells cultured in high glucose and treated with OTX008 (Trotta et al., 2022). On another side, we previously identified the anti-fibrotic properties of another mediator, the flavonoid chrysin (CHR), tested in a rodent model of carbon tetrachloride (CCl_4_)-induced liver fibrosis (Balta et al., 2015; 2018). Therefore, both OTX008 and CHR could have anti-fibrotic effects even in cardiac damage induced by high glucose levels. However, their low solubility in water could affect their *in vivo* administration (Dong et al., 2021; Hermenean et al., 2023).

In this context, we previously formulated a dual-action supramolecular system to improve CHR and OTX008 solubility, aiming at reducing fibrosis in chronic diabetes. Particularly, we first combined CHR with sulfobutylated β-cyclodextrin (SBECD) to improve its limited solubility in water; then we integrated calixarene OTX008, known for its Gal-1 inhibitory properties, into our drug delivery system (Hermenean et al., 2023). This CHR-based supramolecular cyclodextrin-calixarene delivery system was characterized in the context of rat embryonic cardiomyocytes (H9c2) cell viability, ascertaining its safety. Therefore, it could be considered as a promising therapeutic candidate for addressing cardiac fibrosis in chronic diabetes (Hermenean et al., 2023).

To this regard, the present study aimed to explore the potential cardioprotective benefits of the novel CHR-based supramolecular cyclodextrin-calixarene delivery system, by hypothesizing an amplified anti-fibrotic efficacy due to the integration of readily soluble CHR, able to counteract fibrosis, with the selective Gal-1 inhibitor OTX008. Therefore, we investigated the effects of the new drug delivery system in hyperglycemic H9c2 cardiomyocytes and in a mouse model of chronic diabetes, by analyzing the main profibrotic pathways: TGF-β1/SMAD, able to activate myofibroblasts and to increase fibrotic genes, along with ECM deposition (Ma et al., 2018; Saadat et al., 2021); p38, which mediates SMAD-independent TGF-β responses leading to cardiac remodeling, ECM deposition and metalloproteinases (MMPs) modulation (Turner and Blythe, 2019); Erk1/2 mitogen-activated protein kinases (MAPKs), contributing to cardiac fibrosis in diabetic cardiomyopathy (Xu et al., 2016).

## 2 Materials and methods

### 2.1 Materials

OTX008 (Calixarene 0118) was purchased from Selleck Chemicals GmbH, while CHR (5,7-Dihydroxyflavone) from Alfa Aesar (by ThermoFisher Scientific, Kandel, Germany). Sulfobutylated β-cyclodextrin sodium salt (SBECD) (DS∼6) was produced by Cyclolab Ltd. (Budapest, Hungary).

The experimental methods used to obtain the novel CHR complex in OTX-SBECD have been previously detailed and established (Hermenean et al., 2023). Comprehensive phase-solubility evaluations elucidated the mechanisms behind solubility augmentation and complexation. Molecular associations within the cyclodextrin-calixarene-CHR ternary system were assessed via dynamic light-scattering, as well as nuclear magnetic resonance, differential scanning calorimetry, and computational studies, as documented by Hermenean et al. (2023).

### 2.2 *In vitro* setting

As previously described (Hermenean et al., 2023), embryonic rat cardiac H9c2 (2-1) cells (ECACC, United Kingdom) were cultured at 37°C under an atmosphere of 5% CO_2_, in Dulbecco’s modified Eagle’s medium (DMEM; Aurogene, Italy). This growth medium contained 5.5 mM D-glucose, 1% L-Glutamine (L-Glu; AU-X0550 Aurogene, Italy), 10% heat inactivated fetal bovine serum (FBS; AU-S181H Aurogene, Italy) and 1% penicillin/streptomycin (P/S) solution (AU-L0022 Aurogene, Italy). H9c2 cells were seeded at a specific density for each assay before being exposed to NG, high glucose (HG; 33 mM D-glucose) or NG + 27.5 mM mannitol (M; as osmotic control) for 48 h (Hermenean et al., 2023). Cells were then treated in NG or HG medium for 6 days (Hermenean et al., 2023) with the following substances:

- CHR 0.399 mg/mL dissolved in NaCl (CHR);
- SBECD 7.3 m/m% dissolved in NaCl (SBECD);
- SBECD + 0.095 mg/mL CHR dissolved in NaCl (SBECD + CHR);
- as vehicle for OTX008, dimethyl sulfoxide 2.5% (DMSO);
- OTX008 (0.75–1.25–2.50 µM);
- SBECD-OTX008 (2.5–1.25–0.75 µM) dissolved in NaCl (SBECD + OTX);
- SBECD-OTX008 (2.5–1.25–0.75 µM)-CHR dissolved in NaCl (SBECD + OTX + CHR).

Three independent experiments were done, each performed in triplicates (*N* = 3). Cell morphology was observed at the optical microscope.

#### 2.2.1 RNA isolation and real-time quantitative reverse transcription polymerase chain reaction (qRT-PCR)

H9c2 cells were seeded in 6-well plates (1 × 10^5^ cells/well) (Liu et al., 2020), exposed to NG or HG medium for 48 h and then treated for 6 days as previously described. Total RNA was purified from H9c2 lysates with an appropriate isolation kit (217004 Qiagen, Italy). RNA concentration and purity was determined by using the NanoDrop 2000c Spectrophotometer (Thermo Fisher Scientific, Italy). Genomic DNA (gDNA) contaminations were eliminated from RNA samples before the Reverse Transcription (RT) step, carried out on the Gene AMP PCR System 9700 (Applied Biosystems, Italy) by using the QuantiTect Reverse Transcription kit (205311 Qiagen, Italy), according to the protocol “Reverse Transcription with Elimination of Genomic DNA for Quantitative, Real-Time PCR.” The final step for Real Time PCR (qPCR) analysis was carried out in triplicate on the CFX96 Real-time System C1000 Touch Thermal Cycler (Biorad, Italy). This was performed according to the protocol “Two-Step RT-PCR (Standard Protocol),” by using the QuantiTect SYBR Green PCR Kit (204143 Qiagen, Italy) and specific QuantiTect Primer Assays (249900 Qiagen, Italy) for TGF-β1 (QT00187796 Qiagen, Italy), TGFβ receptor 1 (TGFβR1; QT00190953 Qiagen, Italy), TGFβ receptor 2 (TGFβR2; QT00182315 Qiagen, Italy), Erk1 (or MAPK3—QT00176330 Qiagen, Italy) and Erk2 (or MAPK1—QT00190379 Qiagen, Italy) genes. Relative quantization of gene expression was performed by using the 2^^−ΔΔCt^ method, by using rat Glyceraldehyde 3-phosphate dehydrogenase (GAPDH; QT01082004 Qiagen, Italy) as housekeeping control gene.

#### 2.2.2 Enzyme-linked immunosorbent assays (ELISAs)

Cell-biased ELISA assays were performed to analyze the cellular levels of rat p38 MAPK (phosphorylated/total) (CBEL-P38-1 RayBiotech, GA, United States), Smad2 (LS-F1057-1 LSBio, MA, United States) and Smad4 (LS-F2315-1 LSBio, MA, United States) according to the manufacturer’s protocols. Competitive ELISA test was used to quantify the cellular levels of Gal-1 (abx256936 abbexa, United Kingdom), according to the manufacturer’s instructions.

### 2.3 Animals and experimental protocol

Animal experimental procedures were approved by the Ethical Commettee of Vasile Goldis Western University of Arad (Approval number 20, 12/06/2020) and the National Sanitary Veterinary and Food Safety Authority (Certificate number 001/04.02.2021) and were performed according to the guidelines of the Declaration of Helsinki, in compliance with European and national guidelines for research on laboratory animals.

Adult CD1 male mice sourced from the Animal facility of the “Vasile Goldiș” Western University of Arad served as the experimental subjects. These animals were maintained under standardized housing conditions, in compliance with both national and European standards and guidelines. Diabetes was elicited in mice via a single intraperitoneal (i.p.) administration of streptozotocin (STZ) at a dosage of 102 mg/kg body weight. The STZ was freshly prepared in a 50 mM citrate buffer solution (pH 4.5). Post 2 weeks of the STZ administration, fasting blood glucose levels were ascertained. Mice registering blood glucose concentrations exceeding 200 mg/dL were categorized as diabetic and were maintained for a duration of 20 weeks prior to initiating interventions. Post the 20-week period, the chronic diabetic mice, coupled with 10 age-matched healthy counterparts, were randomly assigned into seven distinct groups (*N* = 10 per group):

- Group 1 (Control): Healthy mice serving as the baseline control.
- Group 2 (Diabetes): Chronic diabetic mice, which were euthanized following the 22-week period.
- Group 3 (OTX-SBECD): Chronic diabetic mice at 20 weeks, administered with 5 mg/kg of OTX complexed with SBECD
- Group 4 (OTX-SBECD-CHR): Chronic diabetic mice at 20 weeks, given 5 mg/kg of OTX complexed with SBECD, along with chrysin.
- Group 5 (SBECD-CHR): Chronic diabetic mice at 20 weeks, treated with CHR complexed with SBECD.
- Group 6 (SBECD): Chronic diabetic mice at 20 weeks, receiving the uncomplexed SBECD.

**FIGURE 1:**
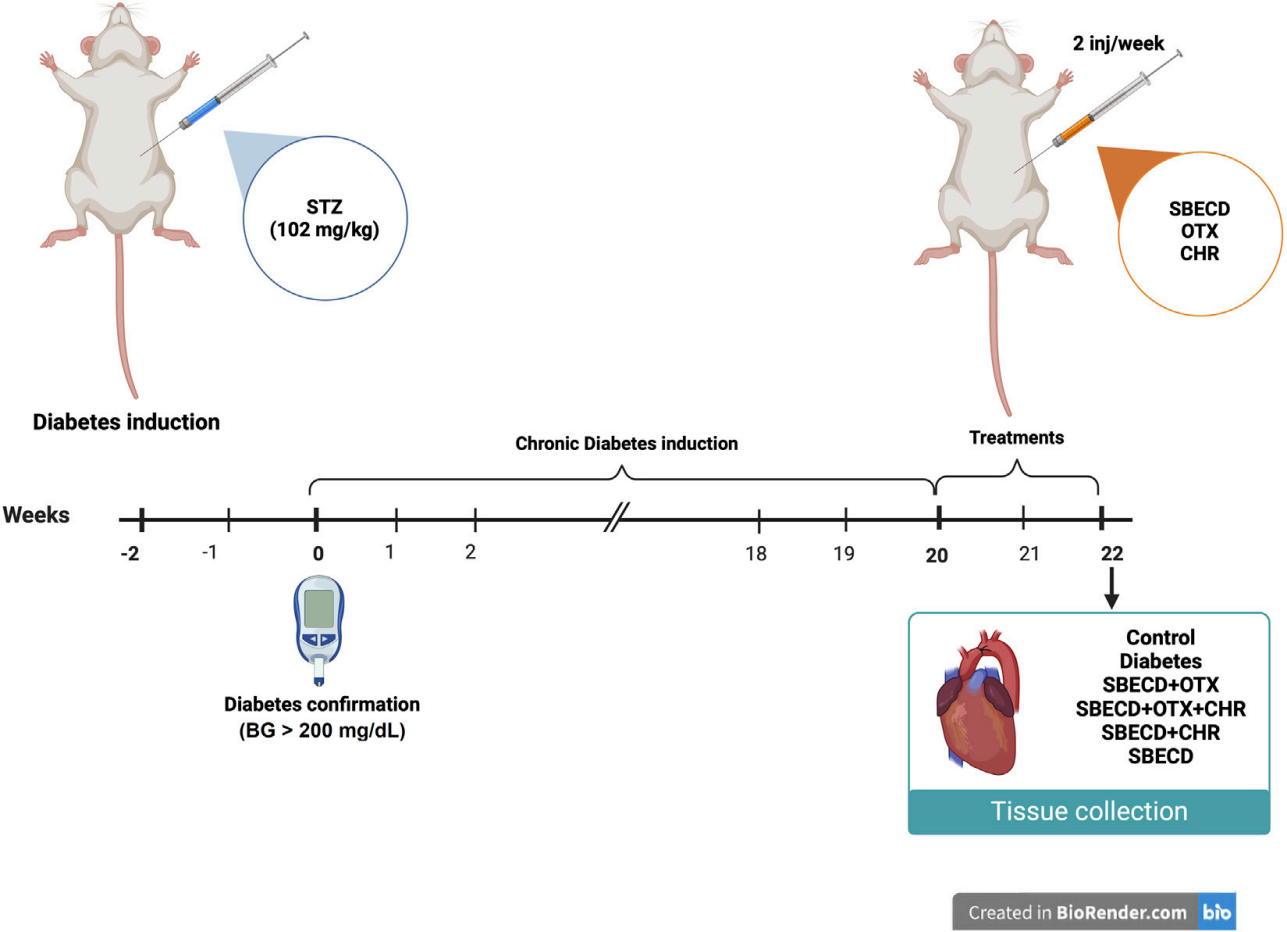
*In vivo* experimental design. This figure was created with BioRender.com.

After 20 weeks of chronic diabetes, treatments were administered 2 times/week for 2 weeks by i.p. injections. Figure 1 provides a schematic representation of the experimental protocol.

#### 2.3.1 Histology

Cardiac specimens were promptly fixed using a 4% paraformaldehyde solution buffered in phosphate buffered saline (PBS) and subsequently embedded in paraffin for processing. Following this, tissue sections were stained with Gomori’s trichrome using the stain kit (38016SS1 Leica, United States). The entire staining procedure adhered closely to the guidelines specified by the Bio-Optica staining kit (Italy). Once stained, the histological slides were examined under an Olympus BX43 microscope (Germany). High-resolution images of the representative sections were captured for documentation and detailed analysis using an Olympus XC30 digital camera (Germany).

#### 2.3.2 Immunohistochemistry

Prior to undertaking immunohistochemistry, heart sections, embedded in paraffin and measuring 5 µm in thickness, underwent deparaffinization and rehydration through established techniques. For antigen detection, sections were treated with primary antibodies—rabbit polyclonal TGF-β1 (sc-146 Santa Cruz Biotechnology, TX, United States), Smad2/3 (sc-133098 Santa Cruz Biotechnology, TX, United States), and α-smooth muscle actin (αSMA, ab32575 abcam, United Kingdom), all at a 1:200 dilution. This was followed by an overnight incubation at 4°C. The Novocastra Peroxidase/DAB kit (Leica Biosystems, Germany) was then employed, consistent with the manufacturer’s directives, to visualize the immunoreactions. For negative controls, primary antibodies were substituted with irrelevant immunoglobulins of the same isotype, and the specimens were observed under bright-field microscopy. Quantitative analysis was performed by determining the ratio of the positively stained area to the total section area using the Image J software.

#### 2.3.3 *In vivo* qRT-PCR evaluations

qRT-PCR was performed to evaluate the mRNA expression levels of TGF-β1, Smad 2/3, Smad 7, Collagen I (Col I), αSMA, MMPs and TIMP metallopeptidase inhibitor 1 (TIMP1). The extraction of total RNA was facilitated using the SV Total RNA isolation kit (Promega, Italy). Following extraction, the integrity and concentration of the RNA samples were gauged via a NanoDrop One spectrophotometer (Thermo Scientific, MA, United States). Subsequently, reverse transcription of the RNA was carried out employing the First Strand cDNA Synthesis Kit (Thermo Scientific, MA, United States). The Maxima SYBR Green/ROX qPCR Master Mix (Life Technologies, CA, United States) was utilized for the RT-PCR, executed on an Mx3000PTM RT-PCR system (Agilent, CA, United States). Every sample underwent triplicate runs for accuracy. Detailed primer sequences are presented in Table 1. Serving as a reference gene, the expression of GAPDH was also gauged, following the identical experimental guidelines. The relative shifts in gene expression were ascertained employing the 2^^−ΔΔCt^ methodology, as documented in reference (Jiang et al., 2021).

**TABLE 1:** Primer sequences for *in vivo* RT-PCR.

Target	Sense	Antisense
TGF-β1	5′ TTT​GGA​GCC​TGG​ACA​CAC​AGT​AC 3′	5′ TGT​GTT​GGT​TGT​AGA​GGG​CAA​GGA 3′
α-SMA	5′ CCGACCGAATGCAGAAG GA 3′	5′ ACA​GAG​TAT​TTG​CGC​TCC​GAA 3′
Smad 2	5′ GTT​CCT​GCC​TTT​GCT​GAG​AC 3′	5′ TCT​CTT​TGC​CAG​GAA​TGC​TT 3′
Smad 3	5′ TGC​TGG​TGA​CTG​GAT​AGC​AG 3′	5′ CTC​CTT​GGA​AGG​TGC​TGA​AG 3′
Smad 7	5′ GCT​CAC​GCA​CTC​GGT​GCT​CA 3′	5′ CCA​GGC​TCC​AGA​AGA​AGT​TG 3′
Col I	5′ CAG​CCG​CTT​CAC​CTA​CAG​C 3′	5′ TTT​TGT​ATT​CAA​TCA​CTG​TCT​TGC​C 3′
MMP1	5′ GCA​GCG​TCA​AGT​TTA​ACT​GGA​A 3′	5′AAC​TAC​ATT​TAG​GGG​AGA​GGT​GT 3′
MMP2	5′ CAG GGA ATG AGT ACT GGG TCT ATT 3′	5′ ACT CCA GTT AAA GGC AGC ATC TAC 3′
MMP3	5′ ACC​AAC​CTA​TTC​CTG​GTT​GCT​GCT 3′	5′ ATG​GAA​ACG​GGA​CAA​GTC​TGT​GGA 3′
MMP9	5′ AAT CTC TTC TAG AGA CTG GGA AGG AG 3′	5′ AGC TGA TTG ACT AAA GTA GCT GGA 3′
Timp1	5′ GGT​GTG​CAC​AGT​GTT​TCC​CTG​TTT 3′	5′TCC​GTC​CAC​AAA​CAG​TGA​GTG​TCA 3′
GAPDH	5′ CGA​CTT​CAA​CAG​CAA​CTC​CCA​CTC​TTC​C-3′	5′ TGG​GTG​GTC​CAG​GGT​TTC​TTA​CTC​CTT 3′

#### 2.3.4 Determination of Gal-1 protein levels

Gal-1 protein levels were assessed in mice cardiac tissues by ELISA assay (EM1051 FineTest, China), according to the manufacturer’s protocol.

### 2.4 Statistical analysis

Data analyses were performed using GraphPad Prism 9.4.0. Results are expressed as mean ± SD. Statistical analysis was performed by employing one-way analysis of variance (ANOVA) with the Bonferroni correction. The strength of association between 2 factors was assessed by Pearson correlation analysis, by determining Pearson correlation coefficient (r). For both ANOVA and Pearson correlation analysis, a *p*-value of <0.05 was considered significant.

## 3 Results

### 3.1 CHR-based supramolecular drug delivery system ameliorates the damaged morphology in H9c2 cells exposed to high glucose

**FIGURE 2:**
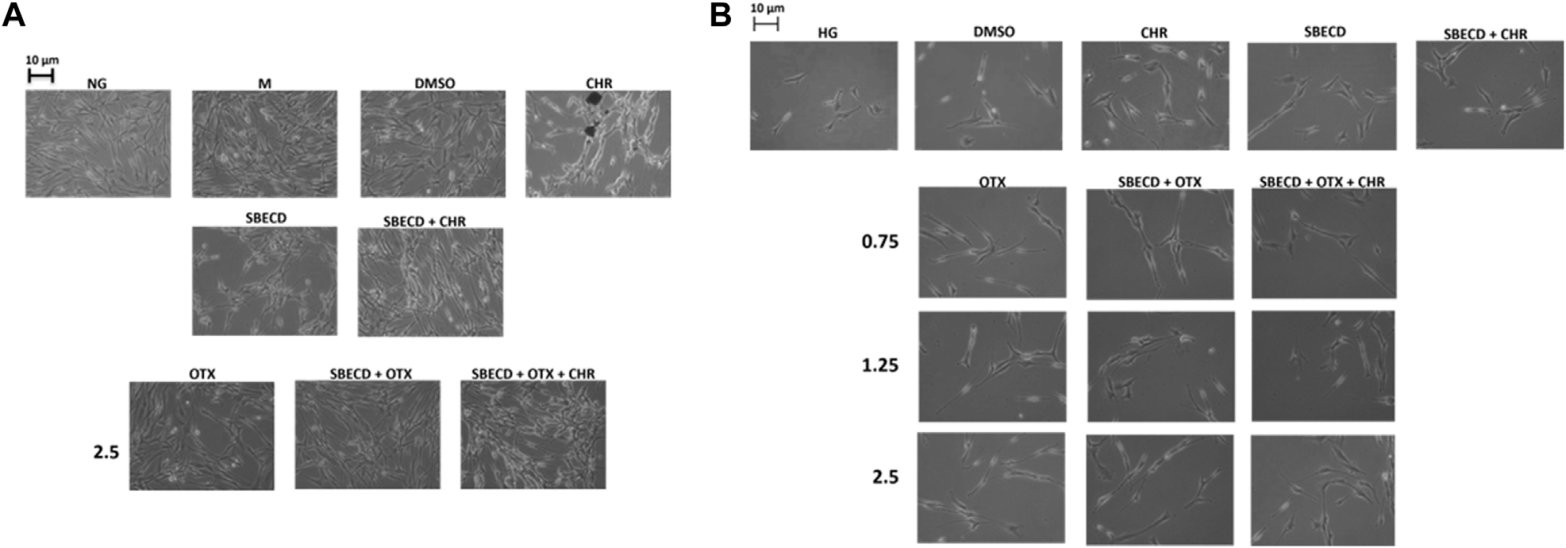
CHR-based supramolecular drug delivery system preserved H9c2 morphology in high glucose. **(A)** Representative optic microscope observations of H9c2 cells cultured in normal glucose (NG) for 48 h and exposed for 6 days to the highest dose (2.5 µM) of OTX008 alone or in the different combinations considered, or **(B)** cultured in high glucose (HG) for 48 h and treated for 6 days with 3 doses (0.75–1.25–2.5 µM) of OTX008 alone or in the different combinations considered. Magnification = ×20; scale bar = 10 µm. *N* = 3 per group (three independent experiments, each performed in triplicate). NG: 5.5 mM D-glucose; M: NG + 27.5 mM mannitol; DMSO: dimethyl sulfoxide 2.5%; CHR: 5,7-Dihydroxyflavone 0.399 mg/mL; SBECD: Sulfobutylated β-cyclodextrin sodium salt 7.3 m/m%; SBECD + CHR: SBECD + 0.095 mg/mL CHR; OTX: calixarene OTX008—Calixarene 0118—(0.75–1.25–2.5): OTX008 (0.75–1.25–2.5 µM); SBECD + OTX: SBECD-OTX008 (2.5–1.25–0.75 µM); SBECD + OTX + CHR: SBECD-OTX008 (2.5–1.25–0.75 µM)-CHR; HG: 33 mM D-glucose.

In normal glucose (NG) condition, the physiological elongated morphology exhibited by H9c2 cells was not affected by the treatments with OTX (2.5 µM) alone or combined with SBECD/SBECD + CHR (Figure 2A). H9c2 morphology was not altered also by the single treatments with CHR, SBECD, SBECD + CHR, DMSO or mannitol (M) (Figure 2A). This was in accordance with our previous data showing a normal viability of these cardiomyocytes in normal glucose when treated with the same compounds (Hermenean et al., 2023). Conversely, H9c2 exposed to high glucose (HG) evidenced a markedly reduced cell viability (Hermenean et al., 2023) and were less elongated, showing a shrunken shape and hypertrophy (Figure 2B). In HG, the treatments with CHR, SBECD and SBECD + CHR did not alter cell viability (Hermenean et al., 2023) and partially recovered the H9c2 damaged cell morphology. This was markedly ameliorated by OTX (0.75–1.25–2.5 µM) alone or in the different formulations with SBECD/SBECD + CHR (Figure 2B), accordingly with the higher increase in cell viability showed by SBECD + OTX and SBECD + OTX + CHR (2.5 µM) (Hermenean et al., 2023).

### 3.2 CHR-based supramolecular drug delivery system reduces the pro-fibrotic pathways in H9c2 cells exposed to high glucose

**FIGURE 3:**
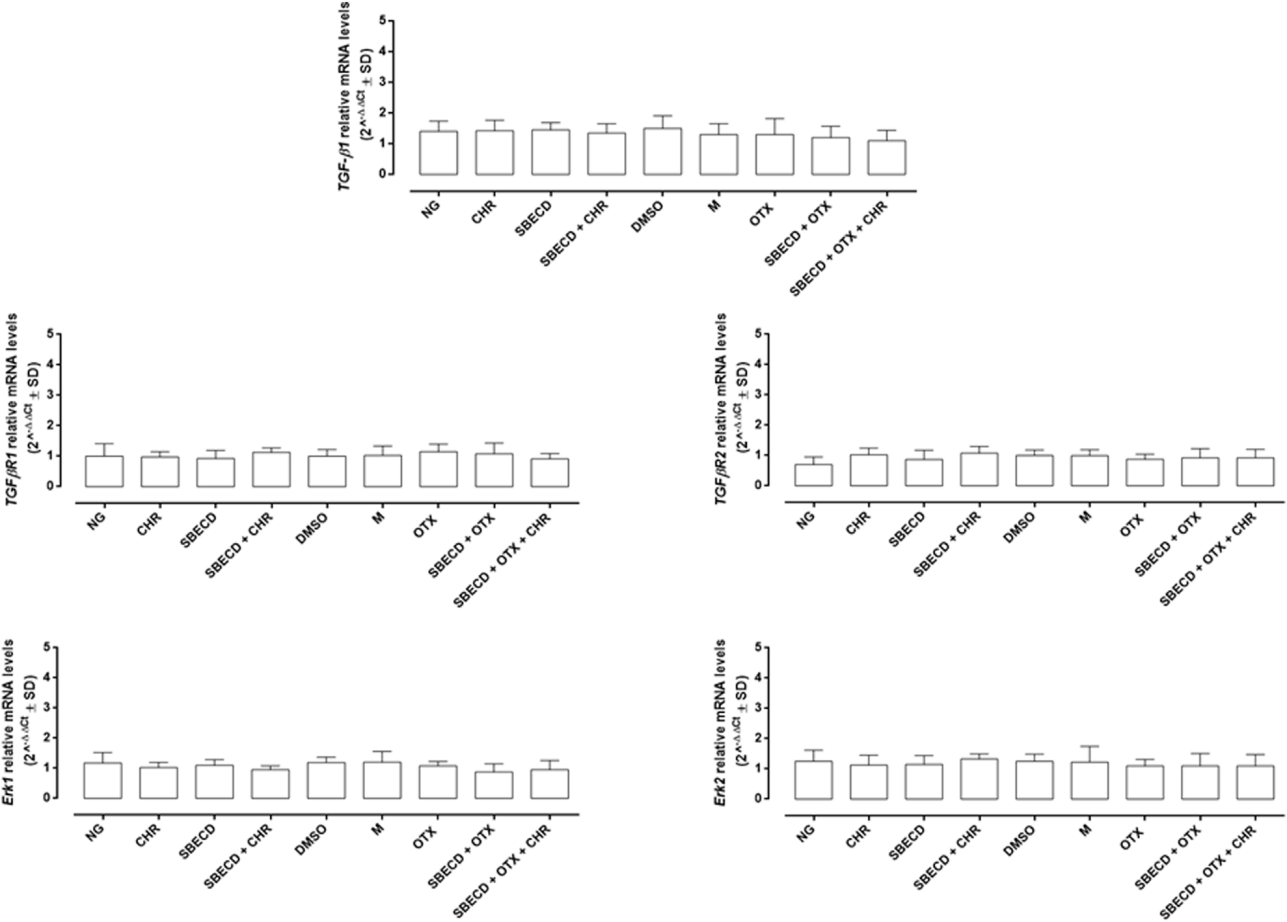
CHR-based supramolecular drug delivery system does not affect TGF-β signaling and MAPKs in H9c2 cardiomyocytes cultured in normal glucose. TGF-β1, TGFβR1, TGFβR2, Erk1 and Erk2 mRNA levels (2^^−ΔΔCt^ ± SD) were determined by qRT-PCR, using GAPDH as gene control. *N* = 3 per group (three independent experiments, each performed in triplicate). NG: 5.5 mM D-glucose; M: NG + 27.5 mM mannitol; DMSO: dimethyl sulfoxide 2.5%; CHR: 5,7-Dihydroxyflavone 0.399 mg/mL; SBECD: Sulfobutylated β-cyclodextrin sodium salt 7.3 m/m%; SBECD + CHR: SBECD + 0.095 mg/mL CHR; OTX: calixarene OTX008—Calixarene 0118—(2.5 µM); SBECD + OTX: SBECD-OTX008 (2.5 µM); SBECD + OTX + CHR: SBECD-OTX008 (2.5 µM)-CHR.

**FIGURE 4:**
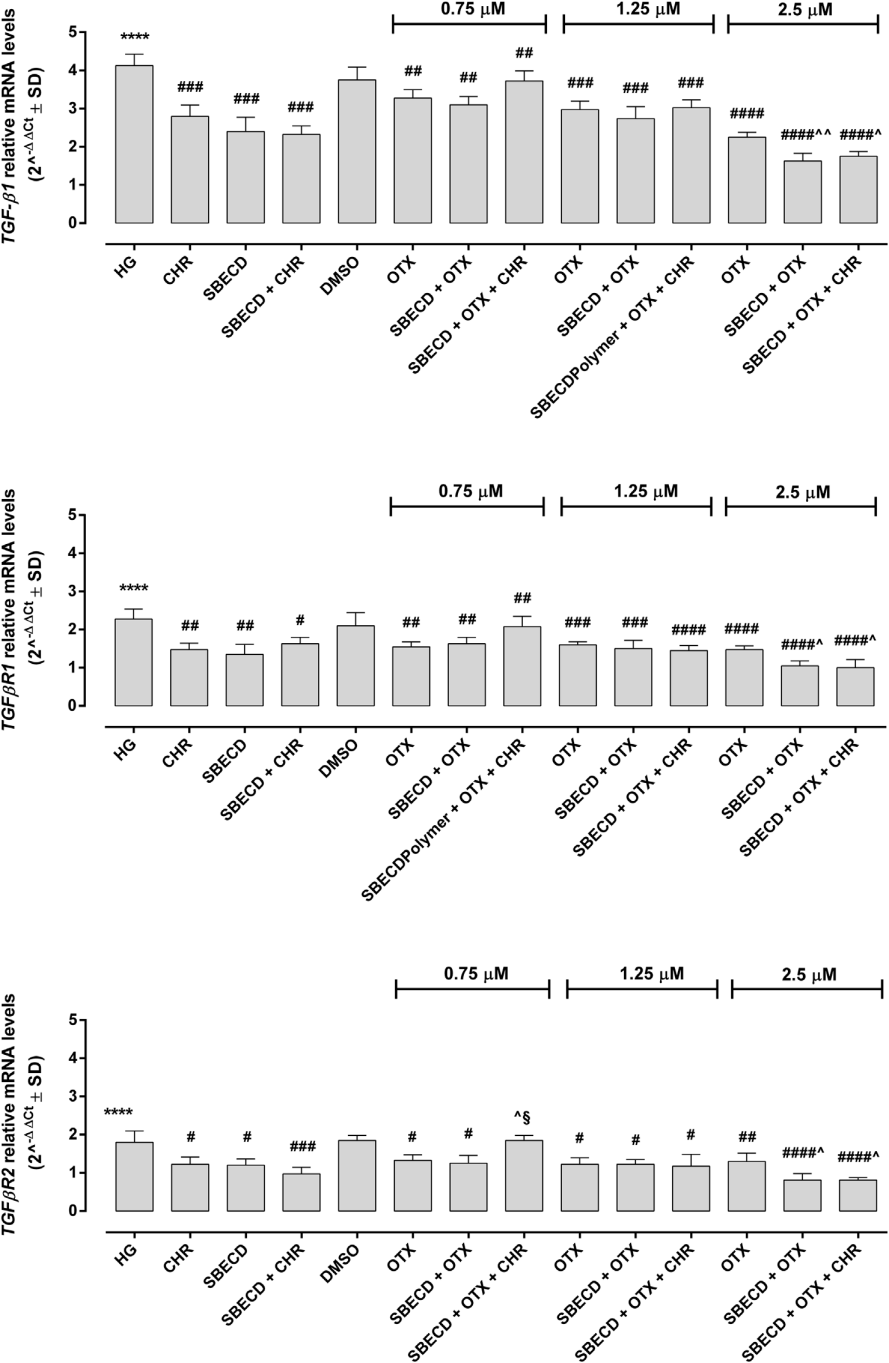
CHR-based supramolecular drug delivery system downregulates TGF-β signaling in H9c2 cardiomyocytes exposed to high glucose. TGF-β1, TGFβR1 and TGFβR2 mRNA levels (2^^−ΔΔCt^ ± SD) were determined by qRT-PCR, using GAPDH as gene control. *N* = 3 per group (three independent experiments, each performed in triplicate). HG: 33 mM D-glucose; M: NG + 27.5 mM mannitol; DMSO: dimethyl sulfoxide 2.5%; CHR: 5,7-Dihydroxyflavone 0.399 mg/mL; SBECD: Sulfobutylated β-cyclodextrin sodium salt 7.3 m/m%; SBECD + CHR: SBECD + 0.095 mg/mL CHR; OTX (0.75–1.25–2.5): calixarene OTX008—Calixarene 0118—(0.75–1.25–2.5 µM); SBECD + OTX (0.75–1.25–2.5): SBECD-OTX008 (0.75–1.25–2.5 µM); SBECD + OTX (0.75–1.25–2.5)+ CHR: SBECD-OTX008 (0.75–1.25–2.5 µM)-CHR. ****p < 0.0001 vs. NG; ^#^ *p* < 0.5, ^##^ *p* < 0.01, ^###^ *p* < 0.001 and ^####^ *p* < 0.0001 vs. HG; ^^^ *p* < 0.05 and ^^ ^^ *p* < 0.01 vs. OTX; ^§^ *p* < 0.05 vs. SBECD + OTX (same dose).

**FIGURE 5:**
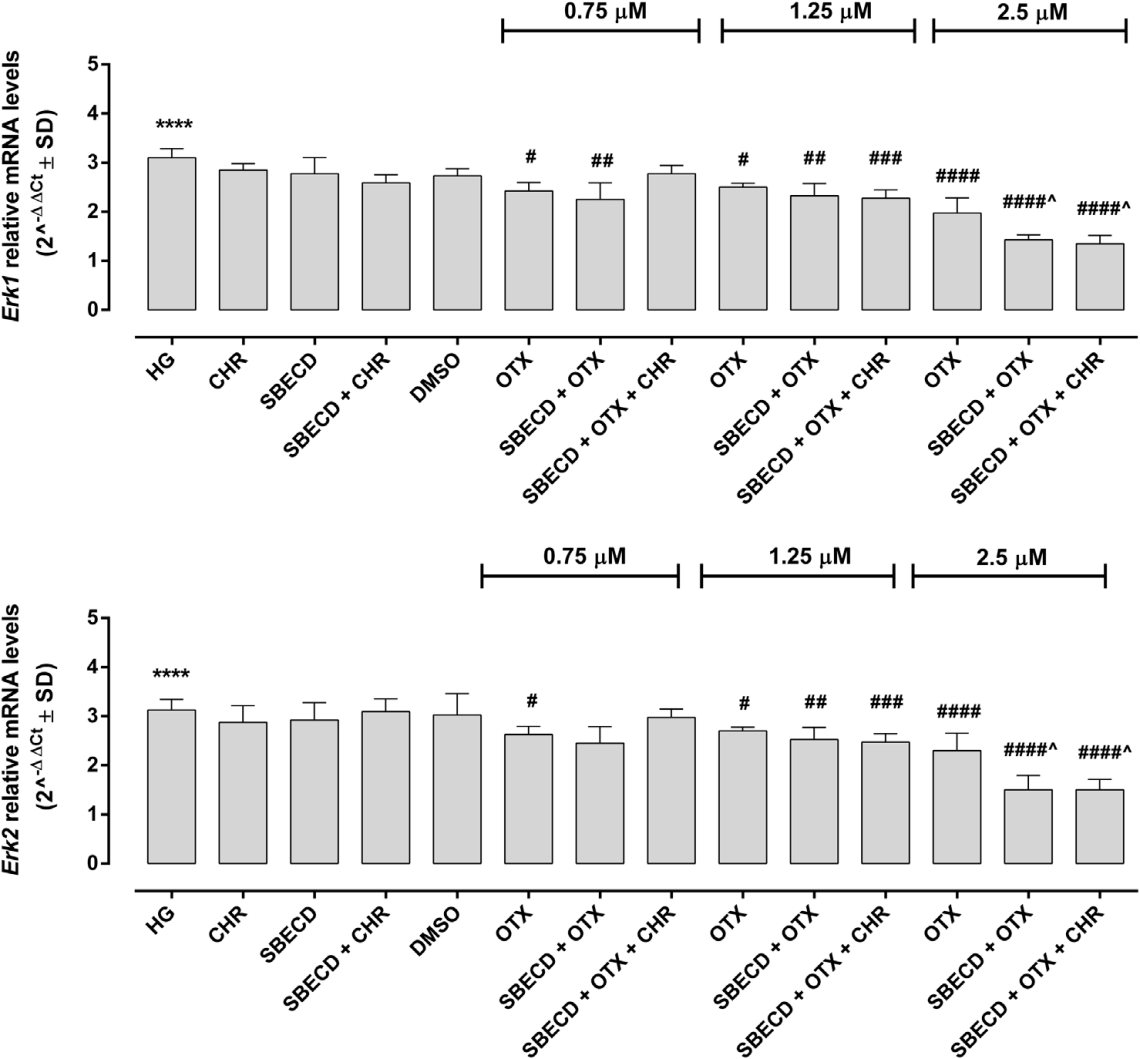
CHR-based supramolecular drug delivery system downregulates MAPKs in H9c2 cardiomyocytes exposed to high glucose. Erk1 and Erk2 mRNA levels (2^^−ΔΔCt^ ± SD) were determined by qRT-PCR, using GAPDH as gene control. *N* = 3 per group (three independent experiments, each performed in triplicate). HG: 33 mM D-glucose; M: NG + 27.5 mM mannitol; DMSO: dimethyl sulfoxide 2.5%; CHR: 5,7-Dihydroxyflavone 0.399 mg/mL; SBECD: Sulfobutylated β-cyclodextrin sodium salt 7.3 m/m%; SBECD + CHR: SBECD + 0.095 mg/mL CHR; OTX (0.75–1.25–2.5): calixarene OTX008—Calixarene 0118—(0.75–1.25–2.5 µM); SBECD + OTX (0.75–1.25–2.5): SBECD-OTX008 (0.75–1.25–2.5 µM); SBECD + OTX (0.75–1.25–2.5)+ CHR: SBECD-OTX008 (0.75–1.25–2.5 µM)-CHR. ****p < 0.0001 vs. NG; ^#^ *p* < 0.5, ^##^ *p* < 0.01, ^###^ *p* < 0.001 and ^####^ *p* < 0.0001 vs. HG; ^*p* < 0.05 vs. OTX (same dose).

The main pro-fibrotic pathways, the canonical (involving TGF-β1, TGFβR1/2, Smad2/4) and non-canonical one (involving p38, Erk1/2 mitogen-activated protein kinases) were not altered in H9c2 cells cultured in normal glucose and exposed to the different treatments (Figure 3; Table 2). Conversely, HG exposure significantly increased both the pro-fibrotic pathways in H9c2 cells (*p* < 0.0001 vs. NG) (Figures 4, 5; Table 3). Interestingly, SBECD, CHR and SBECD + CHR were able to significantly reduce TGF-β1 and TGFβR1/2 expression levels in H9c2 cells exposed to HG, but not the other pro-fibrotic targets. In HG, the three doses of OTX (0.75–1.25 and 2.5 µM) tested alone or in combination with SBECD or SBECD + CHR were able to significantly decrease the canonical and non-canonical fibrotic pathways, but only the two formulations combined with OTX 2.5 µM (SBECD + OTX 2.5 and SBECD + OTX 2.5 + CHR) were able to further decrease the pro-fibrotic pathways compared to same dose of OTX tested alone (Figures 4, 5; Table 3).

**TABLE 2:** Smad2/4 and p38 protein levels in H9c2 cells cultured in normal glucose and exposed to the different treatments.

	NG	CHR	SBECD	SBECD + CHR	DMSO	M	OTX	SBECD + OTX	SBECD + OTX + CHR
Smad2	0.9 ± 0.2	1.1 ± 0.2	1.0 ± 0.2	1.0 ± 0.2	1.0 ± 0.2	0.8 ± 0.3	1.0 ± 0.1	1.0 ± 0.1	1.0 ± 0.3
Smad4	1.0 ± 0.2	1.1 ± 0.2	1.1 ± 0.2	1.0 ± 0.1	1.0 ± 0.2	1.0 ± 0.3	1.1 ± 0.1	1.2 ± 0.3	1.1 ± 0.2
P-p38/p38	1.1 ± 0.2	1.0 ± 0.2	1.0 ± 0.2	1.0 ± 0.2	1.1 ± 0.2	1.0 ± 0.2	1.0 ± 0.2	0.9 ± 0.2	0.9 ± 0.4

Smad2, Smad4 relative protein levels and P-p38/p38 MAPK ratio (optical density values at 450 nm ± SD) were determined by cell-biased ELISA. *N* = 3 per group (three independent experiments, each performed in triplicate). NG: 5.5 mM D-glucose; M: NG + 27.5 mM mannitol; DMSO: dimethyl sulfoxide 2.5%; CHR: 5,7-Dihydroxyflavone 0.399 mg/mL; SBECD: Sulfobutylated β-cyclodextrin sodium salt 7.3 m/m%; SBECD + CHR: SBECD + 0.095 mg/mL CHR; OTX: calixarene OTX008—Calixarene 0118—(2.5 µM); SBECD + OTX: SBECD-OTX008 (2.5 µM); SBECD + OTX + CHR: SBECD-OTX008 (2.5 µM)-CHR.

**TABLE 3:** Smad2/4 and p38 protein levels in H9c2 cells cultured in high glucose and exposed to the different treatments.

	Smad2	Smad4	P-p38/p38
HG	1.9 ± 0.1****	2.3 ± 0.3****	2.5 ± 0.2****
CHR	1.9 ± 0.2	2.2 ± 0.3	2.6 ± 0.2
SBECD	1.7 ± 0.2	2.4 ± 0.4	2.7 ± 0.2
SBECD + CHR	1.8 ± 0.2	2.2 ± 0.2	2.5 ± 0.1
DMSO	1.9 ± 0.2	2.2 ± 0.2	2.4 ± 0.2
OTX (0.75)	1.4 ± 0.1^**#**^	1.6 ± 0.1^**##**^	1.6 ± 0.1^**##**^
SBECD + OTX (0.75)	1.3 ± 0.1^**#**^	1.6 ± 0.2^**##**^	1.7 ± 0.1^**##**^
SBECD + OTX (0.75) + CHR	1.9 ± 0.2^**# ^ §**^	2.0 ± 0.1	2.1 ± 0.1
OTX (1.25)	1.5 ± 0.1^**#**^	1.5 ± 0.2^**####**^	1.8 ± 0.1^**###**^
SBECD + OTX (1.25)	1.4 ± 0.1^**##**^	1.3 ± 0.2^**####**^	1.8 ± 0.1^**####**^
SBECD + OTX (1.25) + CHR	1.5 ± 0.2^**#**^	1.7 ± 0.1^**##**^	2.3 ± 0.2^** ^ §**^
OTX (2.5)	1.5 ± 0.1^**##**^	1.7 ± 0.3^**##**^	1.9 ± 0.1^**###**^
SBECD + OTX (2.5)	1.1 ± 0.1^**#### ^**^	1.3 ± 0.1^**#### ^ ^**^	1.3 ± 0.1^**#### ^ ^**^
SBECD + OTX (2.5) + CHR	1.1 ± 0.1^**#### ^ ^**^	1.7 ± 0.1^**#### ^**^	1.4 ± 0.2^**#### ^**^

Smad2, Smad4 relative protein levels and P-p38/p38 MAPK ratio (optical density values at 450 nm ± SD) were determined by cell-biased ELISA. *N* = 3 per group (three independent experiments, each performed in triplicate). HG: 33 mM D-glucose; M: NG + 27.5 mM mannitol; DMSO: dimethyl sulfoxide 2.5%; CHR: 5,7-Dihydroxyflavone 0.399 mg/mL; SBECD: Sulfobutylated β-cyclodextrin sodium salt 7.3 m/m%; SBECD + CHR: SBECD + 0.095 mg/mL CHR; OTX (0.75–1.25–2.5): calixarene OTX008—Calixarene 0118—(0.75–1.25–2.5 µM); SBECD + OTX (0.75–1.25–2.5): SBECD-OTX008 (0.75–1.25–2.5 µM); SBECD + OTX (0.75–1.25–2.5)+ CHR: SBECD-OTX008 (0.75–1.25–2.5 µM)-CHR. ****p < 0.0001 vs. NG; ^#^ *p* < 0.5, ^##^ *p* < 0.01, ^###^ *p* < 0.001 and ^####^ *p* < 0.0001 vs. HG; ^^^ *p* < 0.05 and ^^ ^^ *p* < 0.01 vs. OTX; ^§^ *p* < 0.05 vs. SBECD + OTX (same dose).

### 3.3 CHR-based supramolecular drug delivery system downregulates the main pro-fibrotic signalling pathways in cardiac tissues

**FIGURE 6:**
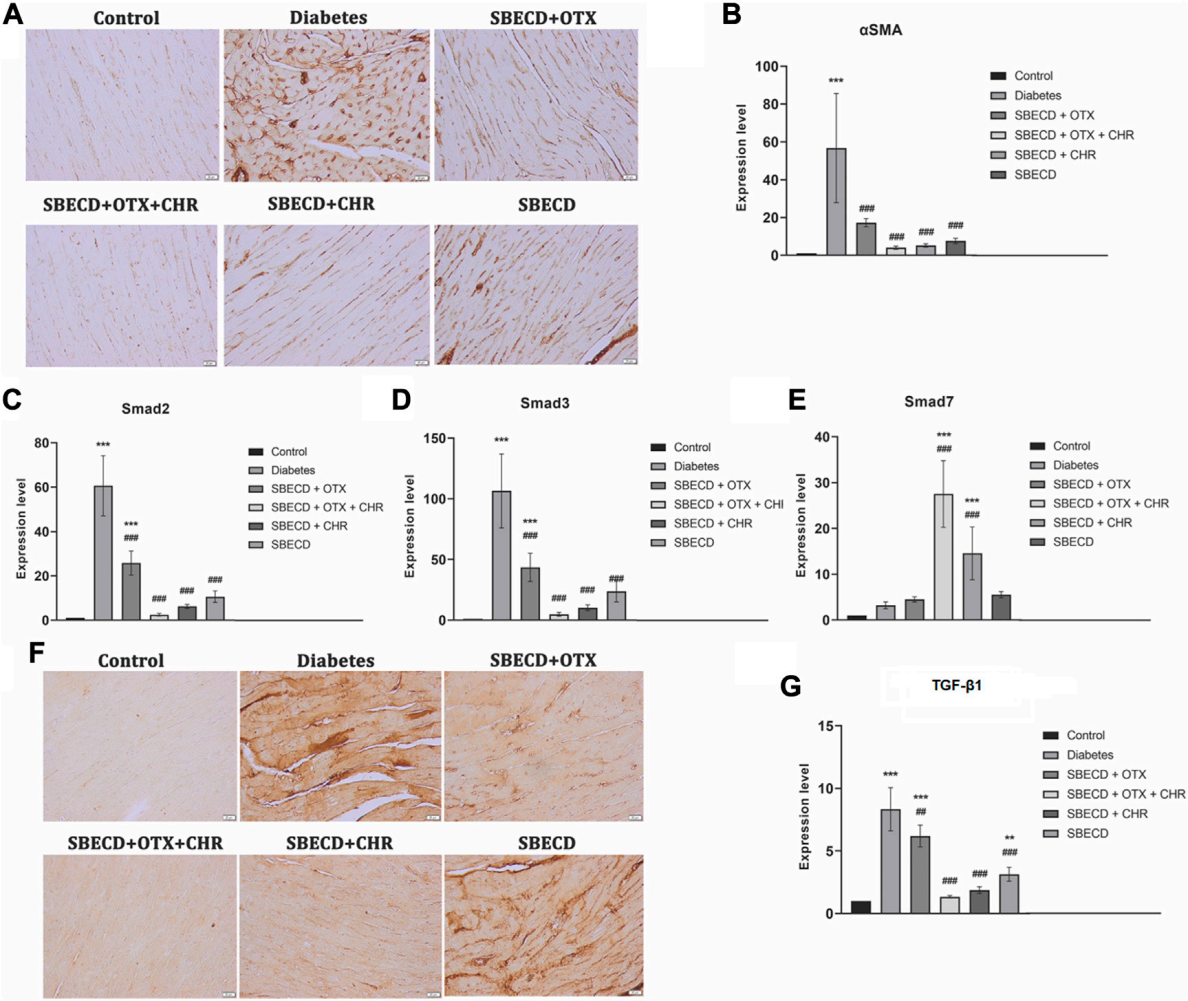
CHR-based supramolecular drug delivery system downregulates the main pro-fibrotic signaling pathways in cardiac tissues. **(A)** Immunohistochemical expression of α-SMA, Scale bar: 20 μm; **(B)** RT-PCR analysis of α-SMA gene expression. **(C)** RT-PCR analysis of Smad2 gene expression; **(D)** RT-PCR analysis of Smad3 gene expression; **(E)** RT-PCR analysis of Smad7 gene expression; **(F)** Immunohistochemical expression of TGF-β1, Scale bar: 20 μm; **(G)** RT-PCR analysis of TGF-β1 gene expression. *N* = 10 mice per group. Control: non-diabetic mice; Diabetes: diabetic mice; SBECD: sulfobutylated β-cyclodextrin; OTX: calixarene OTX008 - Calixarene 0118; CHR: 5,7-Dihydroxyflavone; ***p < 0.001 vs. Control; ^###^ *p* < 0.001 and ^##^ *p* < 0.01 vs. Diabetes.

αSMA gene expression is a marker of activated myofibroblasts in tissue remodeling (Shinde et al., 2017). For the diabetes group, there was a notable increase in αSMA gene expression, as demonstrated by RT-PCR analysis, when compared to the control group (*p* < 0.001). All treatments resulted in significant decreases in this expression relative to the Diabetes group. Among these, the SBECD + OTX + CHR treatment yielded the most substantial decrease, as illustrated in Figure 6. Immunohistochemical studies revealed enhanced staining for the cardiac tissue samples from the Diabetes group, but this expression was almost returned to control levels after SBECD + OTX + CHR treatment.

TGF-β1 plays a crucial role in promoting tissue fibrosis, operating primarily via the phosphorylation of Smad 2/3. In contrast, Smad 7, a Smad inhibitor, works by downregulating Smad 2/3 and targeting the TGF-β1 receptor (Biernacka et al., 2011). In comparison to the control, chronic diabetes led to a significant increase in TGF-β1 gene expression and intense immunopositivity. Treatments with SBECD + OTX, SBECD + OTX + CHR, and SEBCD + CHR lowered TGF-β1 levels by 1.97-fold, 19.11-fold, and 6.62-fold, respectively, when compared to the Diabetes group. The same pattern was obtained for Smad2 and Smad3. In contrast, the mRNA expression of Smad7 was significantly upregulated in SBECD + OTX + CHR group by 12.7-fold compared to diabetes (Figure 6).

### 3.4 CHR-based supramolecular drug delivery system suppresses the secretion and deposition of collagen in cardiac tissues

**FIGURE 7:**
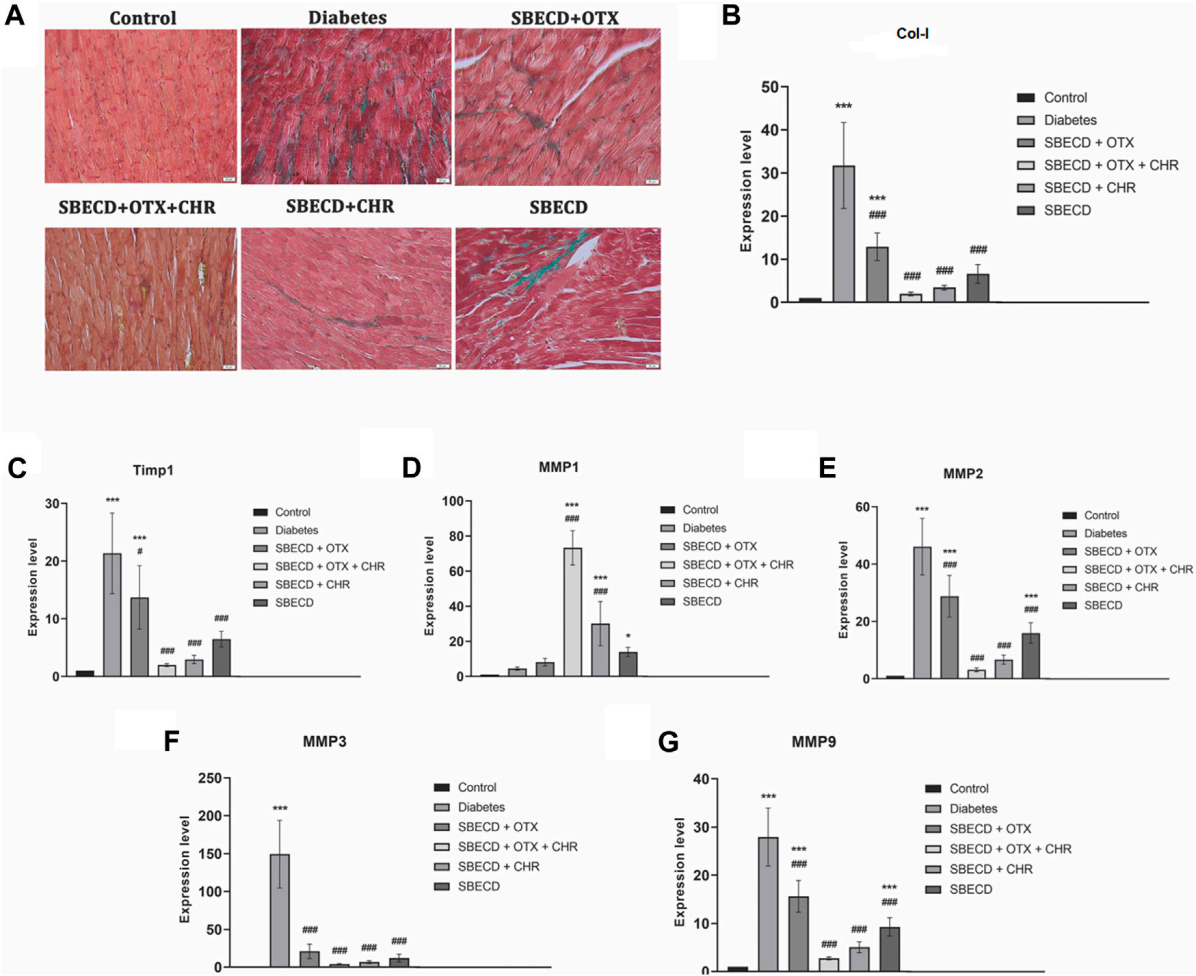
CHR-based supramolecular drug delivery system suppresses the secretion and deposition of collagen in cardiac tissues. **(A)** Collagen staining with Gomori’s Trichrome kit; green—collagen deposition; **(B)** RT-PCR analysis of Col-I gene expression; **(C)** RT-PCR analysis of Timp-1 gene expression; **(D)** RT-PCR analysis of MMP1 gene expression; **(E)** RT-PCR analysis of MMP2 gene expression; **(F)** RT-PCR analysis of MMP3 gene expression; **(G)** RT-PCR analysis of MMP9 gene expression. N = 10 mice per group. Control: non-diabetic mice; Diabetes: diabetic mice; SBECD: sulfobutylated β-cyclodextrin; OTX: calixarene OTX008—Calixarene 0118; CHR: 5,7-Dihydroxyflavone; ***p < 0.001 compared to control; ^#^ *p* < 0.05 and ^###^ *p* < 0.001 vs. Diabetes.

Cardiac tissue displayed an increase in collagen production and accumulation as determined by Gomori’s Trichrome staining in chronic Diabetes group (Figure 7). An RT-PCR examination revealed a significant rise in the expression of Col-1 gene in the diabetes group relative to the control group (*p* < 0.001). Post-treatment, there was a marked reduction in gene expression levels when compared to the diabetes group, with SBECD + OTX + CHR treatment resulting in a decrease by 23.76 times compared to diabetic animals (Figure 7).

TIMP-1 acts as a natural inhibitor, preventing MMPs from breaking down the ECM. To study how the CHR-based cyclodextrin-calixarene supramolecular system affects ECM in the fibrotic heart tissue of diabetic mice, we evaluated the mRNA levels of TIMP-1 and MMPs using RT-PCR. The results indicated that the diabetes group exhibited considerably elevated expression levels of TIMP-1, MMP-2, -3, and -9 genes when contrasted with the control group (*p* < 0.001). The applied treatments effectively reduced these mRNA expressions compared to the diabetes group (*p* < 0.001). However, MMP-1 mRNA expression was notably increased following the treatments relative to the diabetes group (*p* < 0.001). Specifically, in the SBECD + OTX + CHR treatment group, TIMP-1, MMP-2, MMP-3, and MMP-9 expressions dropped approximately by about 29.04, 15.12, 58.77, and 19.41 times, respectively, compared to the diabetes group. Conversely, MMP-1 gene expression raised significantly after treatment with SBECD + OTX + CHR compared to diabetes (*p* < 0.001) (Figure 7).

### 3.5 CHR-based supramolecular drug delivery system modulates Gal-1 levels in H9c2 cells and in cardiac tissues

**FIGURE 8:**
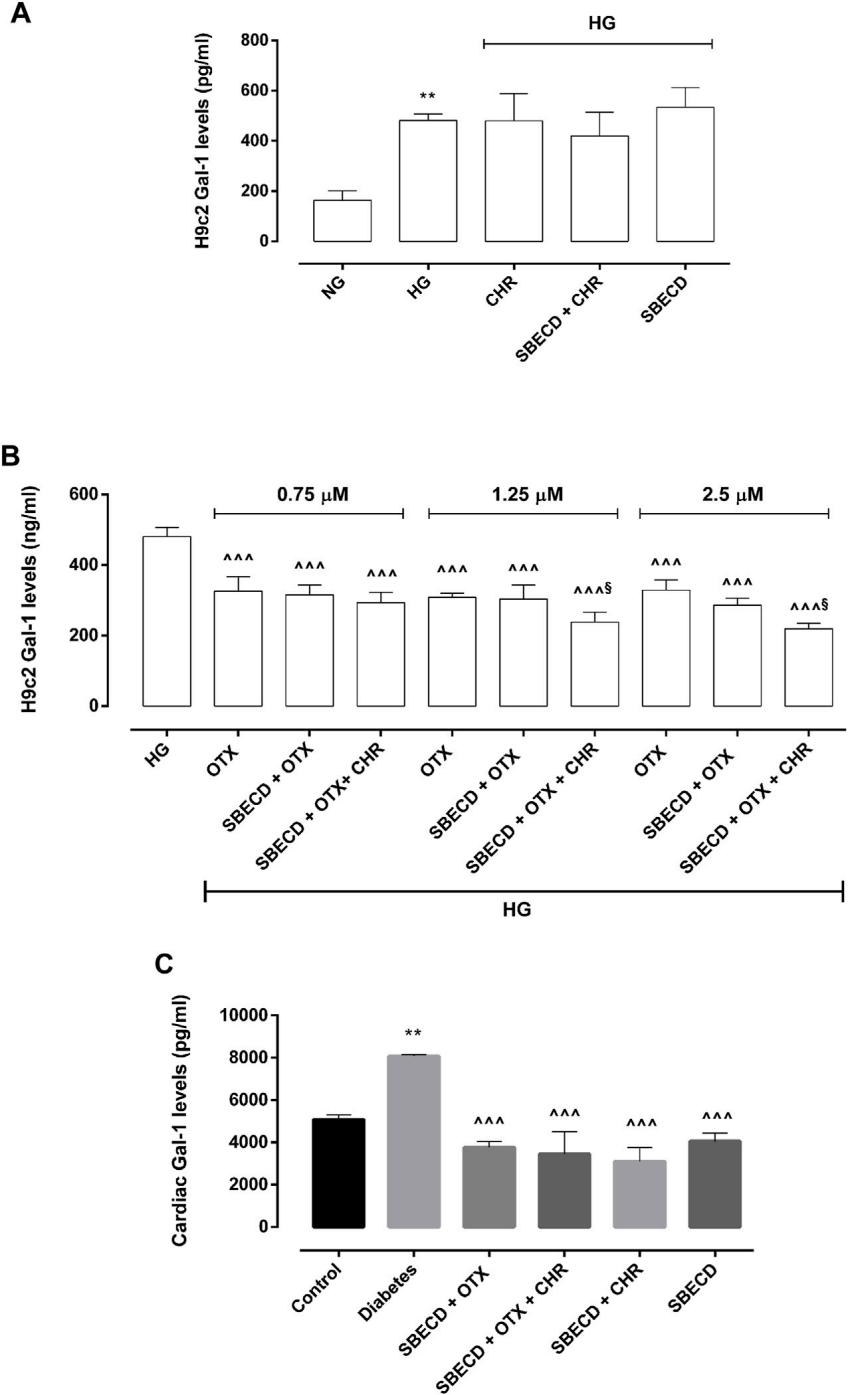
CHR-based supramolecular drug delivery system reduces Gal-1 in H9c2 cells exposed to high glucose and in cardiac tissues. **(A)** Gal-1 protein levels (pg/mL ± SD), determined by ELISA, in H9c2 cells cultured in normal glucose (NG) or high glucose (HG) for 48 h and exposed to CHR, SBECD + CHR and SBECD for 6 days in HG; **(B)** Gal-1 protein levels (pg/mL ± SD) in H9c2 cells cultured in HG for 48 h and treated with OTX008 (0.75–1.25–2.5 µM) alone or combined with SBECD/SBECD + CHR; *N* = 3 per group (three independent experiments, each performed in triplicate). NG: 5.5 mM D-glucose; HG: 33 mM D-glucose; CHR: 5,7-Dihydroxyflavone 0.399 mg/mL; SBECD: Sulfobutylated β-cyclodextrin sodium salt 7.3 m/m%; SBECD + CHR: SBECD + 0.095 mg/mL CHR; OTX: calixarene OTX008—Calixarene 0118—(0.75–1.25–2.5): OTX008 (0.75–1.25–2.5 µM); SBECD + OTX: SBECD-OTX008 (2.5–1.25–0.75 µM); SBECD + OTX + CHR: SBECD-OTX008 (2.5–1.25–0.75 µM)-CHR; **p < 0.01 vs. NG; ^^ ^ ^^ *p* < 0.001 vs. HG; ^§^ *p* < 0.05 vs. OTX; **(C)** Gal-1 protein levels (pg/mL ± SD) determined by ELISA in cardiac tissues. *N* = 10 mice per group. Control: non-diabetic mice; Diabetes: diabetic mice; SBECD: sulfobutylated β-cyclodextrin; OTX: calixarene OTX008—Calixarene 0118; CHR: 5,7-Dihydroxyflavone. **p < 0.01 vs. Control; ^^ ^ ^^ *p* < 0.001 vs. Diabetes.

Gal-1 protein levels were significantly elevated in H9c2 cells exposed to HG (481 ± 26 pg/mL, *p* < 0.01 vs NG) compared to control group (163 ± 38 pg/mL) and were not significantly modulated by CHR (480 ± 108 pg/mL), SBECD + CHR (420 ± 96 pg/mL) and SBECD (534 ± 79 pg/mL) in hyperglycaemic conditions (Figure 7A). All the doses of OTX (0.75–1.25–2.5 µM) alone or in combination with SBECD or SBECD + CHR were able to significantly downregulate Gal-1 protein levels (*p* < 0.001) in H9c2 cells exposed to high glucose (Figure 8B). Moreover, at the doses of 1.25 and 2.5 µM, SBECD + OTX + CHR further reduced Gal-1 protein levels in H9c2 cells compared to OTX at the same doses (*p* < 0.05) (Figure 8B).

Consistent with the *in vitro* findings, after 22 weeks of hyperglycaemia, the cardiac Gal-1 protein levels in chronic diabetes animals showed a significant rise (8,068 ± 71 pg/mL, *p* < 0.01 vs. Control). In contrast, non-diabetic mice exhibited levels of 5,075 ± 233 pg/mL. After 2 weeks of treatments, there was a significant decrease in its levels (*p* < 0.001) (Figure 8C).

## 4 Discussion

Cardiac fibrosis is a defining feature of diabetic cardiomyopathy. This condition is marked by the accumulation of ECM proteins in the cardiac interstitium, leading to perivascular fibrosis and left ventricular (LV) hypertrophy, which frequently results in heart failure (Russo and Frangogiannis, 2016; Jia et al., 2018).

Hyperglycaemia or diabetes has been widely documented to impact the development of cardiac fibrosis, both in preclinical and clinical scenarios. Specifically, rodent models mimicking type I and type II diabetes exhibited a marked cardiomyocyte hypertrophy and myocardial fibrosis, accompanied by the increase of pro-fibrotic genes (Ares-Carrasco et al., 2009; Huynh et al., 2010; 2012; Li et al., 2010; Biernacka et al., 2011; Gonzalez-Quesada et al., 2013; Hao et al., 2015). Similarly, diabetic patients showed extensive interstitial, perivascular and replacement fibrosis (Regan et al., 1977; Kwong et al., 2008; Turkbey et al., 2011), coupled with capillary basement membrane thickening, type I and III collagen accumulation and cardiomyocyte hypertrophy (Fischer et al., 1984; Nunoda et al., 1985; Sutherland et al., 1989; Van Hoeven and Factor, 1990; Shimizu et al., 1993; Kawaguchi et al., 1997; Van Heerebeek et al., 2008; Falcão-Pires et al., 2011). These changes are underlined by the activation of TGF-β pathway following hyperglycaemia (Shamhart et al., 2014), as documented by TGF-β upregulation in the hearts of diabetic rodents exhibiting cardiac fibrosis (Carroll and Tyagi, 2005; Westermann et al., 2007; Senador et al., 2009; Abed et al., 2013).

It is well known that TGF-β isoforms play a central role in the genesis of tissue fibrosis (Biernacka et al., 2011). Specifically, TGF-β1 promotes pro-fibrotic responses in cardiomyocytes through both Smad-dependent and independent signalling (Dobaczewski et al., 2010; Biernacka et al., 2011; Rahaman et al., 2014). Indeed, TGF-β1/Smad pathway plays a crucial role in the activation of myofibroblasts, stimulation of ECM deposition through Col-I synthesis and modulation of MMP activity (Uchinaka et al., 2014; Hanna et al., 2021). On the other hand, TGF-β1 is also able to increasing reactive oxygen species (ROS), consequently activating p38 and Erk1/2 MAPKs (Sano et al., 2001; Wu et al., 2022), leading to collagen deposition in heart tissue (See et al., 2004; Johnston and Gillis, 2022). Importantly, in our prior research, we found a significant reduction in the TGF-β1/Smad pathway in a mouse model of liver fibrosis when treated with CHR (Balta et al., 2015; Balta et al., 2018). This 5,7-dihydroxyflavone was characterized by antioxidant, antitumor, anti-hypercholesterolemic and anti-inflammatory activities (Bae et al., 2011; Brechbuhl et al., 2012; Ali et al., 2014b; Anandhi et al., 2014; Samarghandian et al., 2016; Phan et al., 2021). Our data are in line with other research that identified CHR-mediated inhibition of pro-fibrotic pathways in rat myocardial injury, renal fibrosis and osteoarthritis, by reducing TGF-β1/Smad3, p38, Erk1/2, MMP2 and PERK/TXNIP/NLRP3 signalling (Rani et al., 2015; Nagavally et al., 2021; Ding et al., 2023). Additionally, by using a different pharmacological approach, we demonstrated that TGF-β/Smad pathway can be reduced in human retinal pigment epithelial cells exposed to high glucose by using calixarene OTX008, a selective inhibitor Gal-1 (Trotta et al., 2022). This particular galectin, expressed under normal and pathological conditions, plays a pivotal role in the pathogenesis of fibrosis (Hermenean et al., 2022) and is upregulated in heart failure and acute myocardial infarction (Talman and Ruskoaho, 2016; Seropian et al., 2018). Particularly, Gal-1 is constitutively expressed in cardiomyocytes close to sarcomeric actin, with its expression and secretion increased after cardiac injury promoted by hypoxia, inflammation and fibrosis (Seropian et al., 2018). While its early upregulation can be considered a homeostatic response to prevent cardiac remodeling induced by inflammation, prolonged Gal-1 increase could negatively influence cardiac structure and function (Ou et al., 2021). Interestingly, Gal-1 blocking/silencing by OTX008 has been shown to inhibit TGF-β in various studies, including a cell model of hypoxia-induced pulmonary fibrosis (Kathiriya et al., 2017), a mouse model of liver fibrosis (Jiang et al., 2018), dendritic cells derived from patients with chronic lymphocytic leukaemia (Kostic et al., 2021) and tumour cells (Leung et al., 2014; Koonce et al., 2017).

Worth of note, CHR and Gal-1 have roles that extend beyond influencing pro-fibrotic pathways; they also have implications in the onset and progression of diabetes. Particularly, as evidenced in preclinical studies, CHR seems to attenuate the diabetes-related tissue damage by ameliorating blood glucose levels, insulin resistance and inflammatory state in diabetic animals, by reducing Vascular Endothelial Growth Factor (VEGF) in preclinical models of diabetic retinopathy and by decreasing advanced glycosylation end products (AGEs), TGF-β1/Smad and Col-I deposition in diabetic hearts (Li et al., 2014; Farkhondeh et al., 2019; Zhou et al., 2021a; Salama et al., 2022). This protective effect of CHR is further highlighted by its potential in improving conditions related to diabetes, such as fibrosis (Kang et al., 2023) and cardiometabolic diseases (Talebi et al., 2022). On the other hand, Gal-1 levels have been observed to be elevated in serum of diabetic patients and associated to a reduction of renal function and insulin resistance (Fryk et al., 2016; Drake et al., 2022). Its inhibition, specifically with OTX008, has been proposed in preclinical studies as a possible therapeutic target to treat diabetic renal fibrosis (Al-Obaidi et al., 2019) and proliferative diabetic retinopathy (Abu El-Asrar et al., 2020; Trotta et al., 2022).

Considering their individual protective effects against diabetes-related damage, the co-administration of CHR and calixarene OTX008 presents a potential approach for addressing diabetic fibrosis. However, both CHR and OTX008 showed a lower solubility in water, affecting their bioavailability and absorption (Dong et al., 2021; Hermenean et al., 2023). Indeed, the two compounds are soluble in organic solvents, such as DMSO or dimethylformamide (DMF), which are not suitable for *in vivo* administration at high doses due to their hepatotoxicity (Mathew et al., 1980; Dong et al., 2021; Hermenean et al., 2023). Therefore, we previously developed a dual-action supramolecular drug delivery system, in order to have a water soluble ternary complex. A key step was enhancing CHR’s water solubility. To achieve this, CHR was combined with SBECD, a recognized cyclodextrin derivative known for its safety, polyanionic nature and excellent solubilization properties (Fenyvesi et al., 2020). Then, OTX008 was incorporated in this novel CHR-based supramolecular cyclodextrin-calixarene drug delivery system that synergistically combined cyclodextrin and calixarene. Crucially, safety tests conducted on H9c2 cardiomyocytes revealed no detrimental impacts on cell viability when exposed to this drug delivery system (Hermenean et al., 2023). Moreover, the drug delivery system not only was able to improve H9c2 cell viability in high glucose, but also surpassed the performance of OTX008 when used on its own (Hermenean et al., 2023). This highlights the potential therapeutic promise of this combined approach in diabetic fibrosis treatment.

In this study was characterized the anti-fibrotic effects of the CHR-based supramolecular cyclodextrin-calixarene drug delivery system in H9c2 cells exposed to high glucose. The system demonstrated its strongest anti-fibrotic effects, particularly when paired with the higher dose of OTX008. This lead to a significant reduction of Gal-1 levels, implying a possible modulation of cardiac inflammatory process but primarily leading to the suppression of both canonical and non-canonical profibrotic pathways, in line with previous studies (Leung et al., 2014; Kathiriya et al., 2017; Koonce et al., 2017; Jiang et al., 2018; Kostic et al., 2021; Trotta et al., 2022). Of note, the inhibition of TGF-β1 and its receptors in H9c2 cardiomyocytes could attenuate the fibrotic process by reducing the TGF-β1-induced myofibroblasts activation through Smad2/3 (Yan et al., 2018). Such findings signify the drug delivery system’s ability to counteract the pathological changes instigated by high glucose in cardiomyocytes, as evidenced by the restored morphology of these cells.

Although the CHR-based supramolecular SBECD-calixarene drug delivery system showed were very similar anti-fibrotic actions compared to the combination of OTX008 with SBECD *in vitro*, its higher efficacy in counteracting Gal-1 and the fibrotic pathways was evident in cardiac tissues from mice developing chronic diabetes. Indeed, this dual-action supramolecular drug delivery system led to a marked downregulation of Gal-1, implying an amelioration of cardiac remodeling and inflammatory state, as well as a reduction of profibrotic pathways. To this regard, the decrement of TGF-β1/Smad2/3 pathway in hearts from mice with chronic diabetes may lead to a reduced myofibroblasts transformation and an attenuated cardiac hypertrophic response, though the inhibitions of fibrosis-mediating genes (Fiaschi et al., 2014; Khalil et al., 2017; Yan et al., 2018). Also the increase in cardiac Smad7 levels observed in diabetic mice treated with the SBECD-calixarene drug delivery system could contribute to the reduction of fibrotic process, since Smad7 is a negative regulator of TGF-β signalling in heart, as well as an inhibitor of cardiac remodeling (Chen et al., 2009; Wei et al., 2013). Lastly, the reduction of p38 and Erk1/2 MAPK cardiac levels, associated to abnormal ECM deposition (Tang et al., 2007; Aguilar et al., 2014), confirmed the anti-fibrotic effects of the new drug delivery system treatment.

Along with reduced TGF-β/Smad pathway, a decrease in αSMA levels was observed in diabetic hearts treated with the new drug delivery system. This marker of activated myofibroblasts (Shinde et al., 2017) is higher in cardiomyocytes exposed to high glucose and underwent to myofibroblast conversion, followed by subsequent changes and remodeling in the extracellular matrix (Levick and Widiapradja, 2020). Accordingly, Timp1, an MMP inhibitor stimulated by high glucose levels (Mclennan et al., 2000), was reduced by the treatment with the novel drug delivery system, along with MMP2, 3 and 9 genes, which are typically elevated in diabetic disorders (Zhou et al., 2021b). Under normal physiological circumstances in the heart, MMP1 plays a crucial role in breaking down collagen types I, II, and III, as well as basement membrane proteins (Fan et al., 2012). Our research revealed a decline in MMP1 expression in cardiac tissue subjected to prolonged exposure to high glucose concentrations. This aligns with prior findings where patients with end-stage dilated cardiomyopathy exhibited reduced MMP1 levels in their left ventricular myocardial tissue samples (Thomas et al., 1998). The therapeutic interventions succeeded in elevating MMP1 gene expression, thereby aiding in restoring balance to the matrix components and reverting the cardiac normal architecture. Moreover, the CHR-based supramolecular SBECD-calixarene drug delivery system was the most effective in reducing cardiac Col-I expression and tissue deposition. It is well known that fibrotic heart exhibits Col-I deposition in the cardiac interstitial space, associated with heart dysfunction, dynamic alterations and cardiac remodeling (Kong et al., 2014). Its accumulation induced by high glucose is known to be a factor in myocardial fibrosis, impaired relaxation and mitochondrial degeneration in patients with diabetic cardiomyopathy (Sakakibara et al., 2011; Levick and Widiapradja, 2020). Therefore, Col-I inhibition obtained with the new drug delivery system could be considered a novel strategic therapeutic tool to counteract cardiac fibrosis.

Overall, the CHR-based supramolecular SBECD-calixarene drug delivery system enhanced the solubility and the bioavailability of both CHR and calixarene OTX008. By combining the effects of the two drugs, it showcased a strong anti-fibrotic response in rat cardiomyocytes, as well as in cardiac tissue from mice with chronic diabetes. These evidenced also an improved cardiac tissue remodeling after the treatment with the dual-action supramolecular drug delivery system, which could be considered as a novel putative therapeutic strategy for the treatment of diabetes-induced cardiac fibrosis.

## Data Availability

The original contributions presented in the study are included in the article/supplementary material, further inquiries can be directed to the corresponding author.
